# Supplementary material for: Cpipe: a shared variant detection pipeline designed for diagnostic settings
Source: Genome Med. 2015 Jul 10;7(1):68. doi: 10.1186/s13073-015-0191-x (PMC4515933; doi:10.1186/s13073-015-0191-x)
Supplement: Additional file 2: — An example of the sample summary PDF produced by Cpipe for high level quality control purposes. This example is produced from sequencing reads for 1000 Genomes sample NA12878 over a panel of genes related to cardiomyopathy. [file 13073_2015_191_MOESM2_ESM.pdf]

Sequencing Summary Report for Study NA12878-05

Summary Data

**Batch**	cpipe_paper
**Study ID**	NA12878-05
**Sex**	FEMALE
**Inferred Sex**	FEMALE
**Disease Cohort**	CARDIOM
**Hospital / Institution**	
**Ethnicity**	UNKNOWN
**Prioritzed Genes**	
**Consanguinity Status**	UNKNOWN
**Sample Type (tumor/normal)**	NORMAL
**Sequencing Dates**	
**DNA Collection Dates**	
**Sequencing Machines**	

Coverage Summary

**Reported Mean Coverage**	0.0
**Observed Mean Coverage**	136.84
**Observed Median Coverage**	127
**Total Reads**	91174736
**Fraction on Target**	0.5

Gene Summary

Gene	Category	Perc > 20X	Median	OK?
LMNA		95.0%	145	PASS
TNNT2		100.0%	205	GOOD
ACTN2		96.9%	167	GOOD
VCL		98.7%	185	GOOD
LDB3		92.8%	125	PASS
CSRP3		100.0%	179	GOOD
MYBPC3		77.5%	62	FAIL
ABCC9		99.5%	100	GOOD
MYH7		98.8%	163	GOOD
ACTC1		72.0%	50	FAIL
TPM1		100.0%	184	GOOD

CTF1		82.8%	38	PASS
TCAP		87.1%	81	PASS
TNNI3		100.0%	175	GOOD
DES		94.9%	148	PASS
HOPX		85.2%	107	PASS
SGCD		90.8%	74	PASS
PLN		100.0%	142	GOOD
EMD		99.1%	129	GOOD
TAZ		100.0%	157	GOOD
